# Editorial: Recent advances in cardiotoxicity testing, volume II

**DOI:** 10.3389/fphar.2024.1414373

**Published:** 2024-04-29

**Authors:** Abou-Bakr M. Salama, Tamer M. A. Mohamed

**Affiliations:** Department of Surgery, Baylor College of Medicine, Houston, TX, United States

**Keywords:** heart, cardiac, microphysiological culture systems, hiPSC cardiomyocyte, cardiotoxicity

Over the years, there has been a notable rise in the number of cardiovascular complications associated with various therapeutic interventions, particularly in the domain of cancer treatment (Abdul-Rahman et al., 2023; Pawar et al., 2024). Drug-induced cardiotoxicity poses a significant challenge, often leading to market withdrawals and detrimental health outcomes (Destere et al., 2023). This surge in cardiotoxicity cases underscores the necessity for robust preclinical screening strategies to mitigate risks before advancing to human clinical trials. Traditional screening methods heavily relied on animal models, which, albeit valuable, possess limitations in accurately reflecting human cardiac responses and high financial burden. Consequently, the quest for more reliable and cost-effective alternatives has led to a paradigm shift towards innovative *in vitro* models (Asnani et al., 2021). In recent years, significant strides have been made in leveraging human induced pluripotent stem cell-derived cardiomyocytes (hiPS-CMs), organoids, and human heart slices as promising tools for cardiotoxicity assessment (Miller et al., 2020; Ou et al., 2020; Meki et al., 2021; Rothbauer et al., 2021; Miller et al., 2022; Zhu et al., 2024). These models offer a more physiologically relevant platform, exhibiting high sensitivity and specificity towards diverse therapeutic interventions while faithfully replicating human cardiac physiology and pathophysiology (Wang et al., 2008).

In this Research Topic, “*Recent Advances in Cardiotoxicity Testing, Volume II*,” we present a compilation of eight manuscripts comprising four review articles and four original research articles. Each manuscript delves into the latest breakthroughs in cardiotoxicity testing, encompassing a spectrum of innovative methodologies and insights. This collection serves as a comprehensive resource, shedding light on the cutting-edge strategies and advancements shaping the landscape of cardiotoxicity assessment.

(Altrocchi et al., 2023) described the development and validation of a novel assay utilizing human-induced pluripotent stem cell-derived cardiomyocytes (hiPSC-CMs) to assess both acute and delayed drug-induced cardiotoxic effects of reference compounds with known cardiac outcomes. The assay, conducted on 48-well multielectrode array (MEA) plates, evaluated functional-electrophysiological parameters and viability over 4 days. Testing of various compounds, including tyrosine-kinase inhibitors, classic cardiac toxic drugs, ion-channel trafficking inhibitors, and compounds without known cardiotoxicity, revealed the assay’s ability to recapitulate different cardiotoxicities, such as prolongation of field potential, changes in beating rate, arrhythmic events, and impedance reduction. Additionally, fluorescence- and luminescence-based plate reader assays confirmed cytotoxic effects linked to changes observed in the MEA assay. The study underscores the importance of chronic drug evaluation in identifying cardiotoxic effects, emphasizing the potential of this hiPSC-CMs assay to contribute to early compound de-risking and optimize drug development processes.

Similarly (Arefin et al., 2023), investigated the stability and reproducibility of engineered heart tissues (EHTs) derived from human pluripotent stem cell (hPSC)-differentiated cardiomyocytes for cardiac contractility assays in drug development. Utilizing EHTs from different tissue casting batches and hPSC-cardiomyocyte lines, the researchers assessed contractile outputs using a video-optical assay and compared them with monolayer cultures. Drug-induced variations were tested with compounds known for their cardiac effects or cardiotoxicity, alongside analyses of intracellular calcium transients. Establishing quality control criteria based on excitation-contraction coupling, the study identified suitable EHTs for analysis. Results indicate comparable drug-induced contractile responses between EHTs and monolayers, with a close correlation between contractile output and calcium kinetics. The study emphasizes the importance of reproducibility in assessing drug-induced effects in EHTs, suggesting potential future applications with additional mechanistic criteria for specific contexts of use.

In another context (Advani et al., 2023), aimed at replicating the most common genetic associations of chemotherapy-related cardiotoxicity in the adjuvant NSABP B-31 clinical trial. Using genotyping data from 993 patients, TRPC6 rs77679196 and CBR3 rs1056892 (V244M) were found to be linked to doxorubicin-induced cardiac events in both NCCTG N9831 and NSABP B-31. However, other variants previously reported to be linked to trastuzumab-related decline in LVEF failed to replicate between these studies. In an effort to develop cardioprotective peptides (Zerihun and Qvit, 2023), used computational methods to develop peptides that inhibit mitochondrial fission 1 (Fis1)/mitochondrial dynamics 51 kDa (Mid51) protein-protein interactions to reduce the cellular damage that can lead to cardiotoxicity. They identified two peptides, CVP-241 and CVP-242, and characterized their binding dynamic both *in silico* and *in vitro*.

In this Research Topic (Xiao et al., 2023), provided a bibliometric and knowledge-Map analysis from 2010 to 2022 to spot the light on the hotspots and emerging trends of cardiotoxicity (Lee et al., 2024). provided a perspective regarding the use of iPSC-cardiomyocytes in the preclinical prediction of candidate pharmaceutical toxicity. They explored compared and contrasted the electrophysiology, calcium handling, cellular signaling, contractile machinery, and metabolism between iPSC-CMs and adult CMs. As well they highlighted the methodologies when using iPSC-CMs to ensure a more representative phenotype of the adult human CM. On the other hand (Wu et al., 2023), reviewed the pathogenic mechanism of Immune checkpoint inhibitors-induced cardiotoxicity and evaluated the commonly used cardio-protective drugs. Finally (Zhang et al., 2023), reviewed the gut microbiota product, trimethylamine oxide (TMAO), and its link to cardio pathology, renal pathology, and cardio-renal syndrome.

As this field of research is evolving rapidly, the present volume of the Research Topic provides an update on the mechanisms of chemotherapy-induced cardiotoxicities, genetic variants related to them, possible modifiers and targets including peptides and metabolites, and testing platforms.

## References

[B1] Abdul-Rahman T. Dunham A. Huang H. Bukhari S. M. A. Mehta A. Awuah W. A. (2023). Chemotherapy induced cardiotoxicity: a state of the art review on general mechanisms, prevention, treatment and recent advances in novel therapeutics. Curr. Problems Cardiol. 48 (4), 101591. 10.1016/j.cpcardiol.2023.101591 36621516

[B2] Advani P. P. Ruddy K. J. Herrmann J. Ray J. C. Craver E. C. Yothers G. (2023). Replication of genetic associations of chemotherapy-related cardiotoxicity in the adjuvant NSABP B-31 clinical trial. Front. Oncol. 13, 1139347. 10.3389/fonc.2023.1139347 37305569 PMC10248403

[B3] Altrocchi C. Van Ammel K. Steemans M. Kreir M. Tekle F. Teisman A. (2023). Evaluation of chronic drug-induced electrophysiological and cytotoxic effects using human-induced pluripotent stem cell-derived cardiomyocytes (hiPSC-CMs). Front. Pharmacol. 14, 1229960. 10.3389/fphar.2023.1229960 37492082 PMC10364322

[B4] Arefin A. Mendoza M. Dame K. Garcia M. I. Strauss D. G. Ribeiro A. J. S. (2023). Reproducibility of drug-induced effects on the contractility of an engineered heart tissue derived from human pluripotent stem cells. Front. Pharmacol. 14, 1212092. 10.3389/fphar.2023.1212092 37469866 PMC10352809

[B5] Asnani A. Moslehi J. J. Adhikari B. B. Baik A. H. Beyer A. M. de Boer R. A. (2021). Preclinical models of cancer therapy–associated cardiovascular toxicity: a scientific statement from the American heart association. Circulation Res. 129 (1), e21–e34. 10.1161/RES.0000000000000473 33934611 PMC8423100

[B6] Destere A. Merino D. Lavrut T. Rocher F. Viard D. Drici M.-D. (2023). Drug-induced cardiac toxicity and adverse drug reactions, a narrative review. *Therapies* . 10.1016/j.therap.2023.10.008 37957054

[B7] Lee T. Y. T. Coles J. G. Maynes J. T. (2024). iPSC-cardiomyocytes in the preclinical prediction of candidate pharmaceutical toxicity. Front. Pharmacol. 15, 1308217. 10.3389/fphar.2024.1308217 38482053 PMC10933010

[B8] Meki M. H. Miller J. M. Mohamed T. M. A. (2021). Heart slices to model cardiac physiology. Front. Pharmacol. 12, 617922. 10.3389/fphar.2021.617922 33613292 PMC7890402

[B9] Miller J. M. Meki M. H. Elnakib A. Ou Q. Abouleisa R. R. E. Tang X.-L. (2022). Biomimetic cardiac tissue culture model (CTCM) to emulate cardiac physiology and pathophysiology *ex vivo* . Commun. Biol. 5 (1), 934. 10.1038/s42003-022-03919-3 36085302 PMC9463130

[B10] Miller J. M. Meki M. H. Ou Q. George S. A. Gams A. Abouleisa R. R. E. (2020). Heart slice culture system reliably demonstrates clinical drug-related cardiotoxicity. Toxicol. Appl. Pharmacol. 406, 115213. 10.1016/j.taap.2020.115213 32877659 PMC7554180

[B11] Ou Q. Abouleisa R. R. E. Tang X.-L. Juhardeen H. R. Meki M. H. Miller J. M. (2020). Slicing and culturing pig hearts under physiological conditions. JoVE J. Vis. Exp. (157), e60913. 10.3791/60913 PMC7388059 32250357

[B12] Pawar V. Wankhede Y. Kaur S. Pawar B. Vasdev N. Gupta T. (2024). Drug-induced cardiotoxicity. Public Health Toxicol. Issues Drug Res. 2, 253–282. 10.1016/b978-0-443-15842-1.00024-7

[B13] Rothbauer M. Bachmann B. E. M. Eilenberger C. Kratz S. R. A. Spitz S. Höll G. (2021). A decade of organs-on-a-chip emulating human physiology at the microscale: a critical status report on progress in toxicology and pharmacology. Micromachines 12 (5), 470. 10.3390/mi12050470 33919242 PMC8143089

[B14] Wang D. Patel C. Cui C. Yan G.-X. (2008). Preclinical assessment of drug-induced proarrhythmias: role of the arterially perfused rabbit left ventricular wedge preparation. Pharmacol. Ther. 119 (2), 141–151. 10.1016/j.pharmthera.2008.02.009 18423604

[B15] Wu Y. Xu Y. Xu L. Peng H. Gao A. Wang J. (2023). Effects of Shenmai injection against chronic heart failure: a meta-analysis and systematic review of preclinical and clinical studies. Front. Pharmacol. 14, 1338975. 10.3389/fphar.2023.1338975 38385058 PMC10880451

[B16] Xiao D. Li J. Liu Y. Wang T. Niu C. Zhuang R. (2023). Emerging trends and hotspots evolution in cardiotoxicity: a bibliometric and knowledge-Map analysis from 2010 to 2022. Front. Cardiovasc. Med. 10, 1089916. 10.3389/fcvm.2023.1089916 36960468 PMC10029978

[B17] Zerihun M. Qvit N. (2023). Selective inhibitors targeting Fis1/Mid51 protein-protein interactions protect against hypoxia-induced damage in cardiomyocytes. Front. Pharmacol. 14, 1275370. 10.3389/fphar.2023.1275370 38192411 PMC10773907

[B18] Zhang J. Zhu P. Li S. Gao Y. Xing Y. (2023). From heart failure and kidney dysfunction to cardiorenal syndrome: TMAO may be a bridge. Front. Pharmacol. 14, 1291922. 10.3389/fphar.2023.1291922 38074146 PMC10703173

[B19] Zhu Y. Yang S. Zhang T. Ge Y. Wan X. Liang G. (2024). Cardiac organoids: a 3D technology for disease modeling and drug screening. Curr. Med. Chem. 31. 10.2174/0929867331666230727104911 37497713

